# Direct adenylation from 5′-OH-terminated oligonucleotides by a fusion enzyme containing Pfu RNA ligase and T4 polynucleotide kinase

**DOI:** 10.1093/nar/gkac604

**Published:** 2022-07-12

**Authors:** Zhengquan Yang, Chengliang Zhang, Guojun Lian, Shijie Dong, Menghui Song, Hengrong Shao, Jingmei Wang, Tao Zhong, Zhenni Luo, Shengnan Jin, Chunming Ding

**Affiliations:** Key Laboratory of Laboratory Medicine, Ministry of Education of China, School of Laboratory Medicine and Life Sciences, Wenzhou Medical University, Wenzhou, Zhejiang, 325035, China; Key Laboratory of Laboratory Medicine, Ministry of Education of China, School of Laboratory Medicine and Life Sciences, Wenzhou Medical University, Wenzhou, Zhejiang, 325035, China; Department of Clinical Laboratory, Kunming Third People's Hospital, Kunming, Yunnan, 650041, China; Key Laboratory of Laboratory Medicine, Ministry of Education of China, School of Laboratory Medicine and Life Sciences, Wenzhou Medical University, Wenzhou, Zhejiang, 325035, China; Key Laboratory of Laboratory Medicine, Ministry of Education of China, School of Laboratory Medicine and Life Sciences, Wenzhou Medical University, Wenzhou, Zhejiang, 325035, China; Key Laboratory of Laboratory Medicine, Ministry of Education of China, School of Laboratory Medicine and Life Sciences, Wenzhou Medical University, Wenzhou, Zhejiang, 325035, China; Key Laboratory of Laboratory Medicine, Ministry of Education of China, School of Laboratory Medicine and Life Sciences, Wenzhou Medical University, Wenzhou, Zhejiang, 325035, China; Key Laboratory of Laboratory Medicine, Ministry of Education of China, School of Laboratory Medicine and Life Sciences, Wenzhou Medical University, Wenzhou, Zhejiang, 325035, China; Key Laboratory of Laboratory Medicine, Ministry of Education of China, School of Laboratory Medicine and Life Sciences, Wenzhou Medical University, Wenzhou, Zhejiang, 325035, China; Key Laboratory of Laboratory Medicine, Ministry of Education of China, School of Laboratory Medicine and Life Sciences, Wenzhou Medical University, Wenzhou, Zhejiang, 325035, China; Key Laboratory of Laboratory Medicine, Ministry of Education of China, School of Laboratory Medicine and Life Sciences, Wenzhou Medical University, Wenzhou, Zhejiang, 325035, China; Key Laboratory of Laboratory Medicine, Ministry of Education of China, School of Laboratory Medicine and Life Sciences, Wenzhou Medical University, Wenzhou, Zhejiang, 325035, China

## Abstract

5′-Adenylated oligonucleotides (AppOligos) are widely used for single-stranded DNA/RNA ligation in next-generation sequencing (NGS) applications such as microRNA (miRNA) profiling. The ligation between an AppOligo adapter and target molecules (such as miRNA) no longer requires ATP, thereby minimizing potential self-ligations and simplifying library preparation procedures. AppOligos can be produced by chemical synthesis or enzymatic modification. However, adenylation via chemical synthesis is inefficient and expensive, while enzymatic modification requires pre-phosphorylated substrate and additional purification. Here we cloned and characterized the Pfu RNA ligase encoded by the *PF0353* gene in the hyperthermophilic archaea *Pyrococcus furiosus*. We further engineered fusion enzymes containing both Pfu RNA ligase and T4 polynucleotide kinase. One fusion enzyme, 8H-AP, was thermostable and can directly catalyze 5′-OH-terminated DNA substrates to adenylated products. The newly discovered Pfu RNA ligase and the engineered fusion enzyme may be useful tools for applications using AppOligos.

## INTRODUCTION

5′-Adenylated oligonucleotide (AppOligo) linkers or DNA adapters are used in next-generation sequencing (NGS) of small RNAs and single-stranded DNAs (ssDNAs) during library preparation (1–3). For small RNA sequencing library preparation, 5′ pre-adenylated adapter is ligated to the 3′ end of RNA molecules using either T4 RNA ligase 1 or a truncated T4 RNA ligase 2 without adding ATP, thereby removing the need to dephosphorylate the substrate RNA to avoid unwanted self-ligation byproducts (1,2,4,5).

AppOligos can be produced by either chemical synthesis or enzymatic modification (5–8). The chemical synthesis method uses the reaction between a 5′-phosphorylated oligonucleotide and the pre-activated adenosine-5′-monophosphate (AMP) in solution or on a solid support (8,9), a process with poor conversion efficiency while also requiring the block of the 3′ end of the substrate to prevent self-ligation. As such, additional purification by methods such as high-performance liquid chromatography (HPLC) and polyacrylamide gel electrophoresis (PAGE) is necessary to remove the unconverted substrate (6).

Enzymatic modification relies on the ability of DNA or RNA ligases to transfer the AMP residue to the 5′-phosphate of oligonucleotides (10–12). Adenylated DNA or RNA is an intermediate in the ligation reaction which usually does not accumulate during ligation (7,8). The current enzymatic approach to produce AppOligos requires an interruption of the ligation reaction to generate an AppOligo intermediate by either not providing the acceptor or introducing a template containing a mismatched nucleotide opposite to the 5′-phosphoryl DNA donor site (13,14). An alternative enzymatic approach is to use template-independent RNA ligases or cyclases, such as Mth RNA ligase (Mth Rnl), TS2126 RNA ligase or *Escherichia coli* RNA 3′-phosphate cyclase (RtcA), to catalyze phosphorylated DNA or RNA to adenylated products directly (12,15,16). However, these reactions require pre-phosphorylated oligonucleotides and a high concentration of ATP to prevent template circularization.

In the present study, we identified and characterized an enzyme named Pfu RNA ligase (Pfu Rnl) from *Pyrococcus furiosus*. Pfu Rnl was thermostable and showed high adenylation efficiency for phosphorylated DNA or RNA oligonucleotide substrate while having no noticeable DNA ligation activity. We also engineered fusion enzymes by fusing Pfu Rnl with T4 polynucleotide kinase (T4 PNK). The fusion enzymes have both phosphorylation and adenylation activity to convert 5′-OH-terminated DNA oligonucleotides to adenylated products in a single reaction with high efficiency.

## MATERIALS AND METHODS

### Oligonucleotides

5′-Phosphorylated or 5′-OH-terminated oligonucleotides were purchased from Genscript (Nanjing, China). Sequences of oligonucleotides with different modifications used for enzyme activity analysis are listed in Supplementary Table S1.

### Protein structure prediction and superposition

The Pfu Rnl amino acid sequence was submitted to the SWISS-MODEL online server (Version 2022-03-23) and modeled against Pab1020 RNA ligase (PDB: 2VUG) (17,18). The modeled structure of Pfu Rnl was superposed to Mth Rnl (PDB: 5D1P) using PyMol (The PyMOL Molecular Graphics System, Version 2.0 Schrödinger, LLC.) (19).

### Plasmids for protein expression

The coding sequence for Pfu Rnl was codon optimized and cloned into the pET28a expression vector (Novagen Inc.) containing an 8× His tag at the N-terminus. The coding sequence for T4 PNK was codon optimized with a 6× His tag at the N-terminus in the pET28a vector. For T4 PNK and Pfu Rnl fusion proteins, a Gly/Ser (GS)-rich linker sequence was inserted between the two enzymes (20,21). The expression plasmids for the fusion proteins were constructed using seamless cloning with the ClonExpress II One Step Cloning Kit (Vazyme). For example, for 8H-AP plasmid construction, the pET28a expression vector with full-length Pfu Rnl was amplified with inverse PCR using Pfu_F and Pfu_R PCR primers (22). The full-length T4 PNK coding sequence was amplified using PNK_ins_F and PNK_ins_R primers. PCR products were purified after agarose gel electrophoresis. Equal molar amounts of T4 PNK PCR products were mixed with the Pfu Rnl plasmid PCR products and then cloned with the ClonExpress II One Step Cloning Kit. All plasmids were verified by Sanger sequencing. Primers used for plasmid construction are listed in Supplementary Table S2.

### Protein expression and purification

For Pfu Rnl expression, a 1 l culture of transformed *E. coli* BL21(DE3) was incubated at 37°C in Luria–Bertani medium containing 50 mg kanamycin/l until the *A*_600_ reached 0.6–0.8, before adding isopropyl-β-d-1-thiogalactopyranoside (IPTG) to a final concentration of 1 mM to induce expression (19). Cells were incubated for an additional 16 h at 25°C and harvested by centrifugation at 5000 rpm. The cells were resuspended in His Binding buffer (20 mM NaH_2_PO_4_, 300 mM NaCl, 20 mM imidazole, pH 7.6) and sonicated with 30 s continuous pulsing at 4°C for 30 min on a SCIENTZ-950E ultrasonic disruptor (SCIENTZ Biotechnology Co., Ltd). The lysate was centrifuged at 12 000 *g* for 20 min at 4°C and the soluble fraction was obtained. After filtering with a 0.22 μm filter, the supernatant was loaded onto the HisTrap FF column (GE Healthcare) with the AKTA Purifier L25 (GE Healthcare) and eluted stepwise with 10, 20, 40 and 100% His Elution buffer (20 mM NaH_2_PO_4_, 300 mM NaCl, 500 mM imidazole, pH 7.6). Eluted protein was diluted 10-fold with Heparin buffer A (20 mM NaH_2_PO_4_, 20 mM NaCl, pH 7.6) and purified with the HiTrap Heparin HP affinity column (GE Healthcare). The protein was then eluted stepwise with 20, 70 and 100% Heparin buffer B (20 mM NaH_2_PO_4_, 1 M NaCl, pH 7.6). The collected fractions were concentrated with an Amicon Ultra-15 centrifugal filter (Merck Millipore), quantified using the Pierce BCA protein assay kit (Thermo Fisher Scientific) and analyzed with SDS–polyacrylamide gel electrophoresis (SDS–PAGE). The protein solution was finally mixed with an equal volume of glycerol and stored at –20°C (23). T4 PNK and the fusion enzymes were expressed and purified using the same method as described above.

### Gel filtration

Standard molecular weight proteins from the high molecular weight Gel Filtration Calibration Kit (GE Healthcare) were dissolved with the running buffer (50 mM phosphate, 150 mM NaCl, pH 7.0) as described in the manual. Briefly, for standard proteins, 0.5 ml of each standard protein was loaded onto the HiLoad 26/600 Superdex 200 pg column (GE Healthcare) with a recommended flow rate of 2.6 ml/min in the AKTA purifier L25. Blue Dextran 2000 was used to detect the void volume. UV peaks of each protein were used to calculate the elution volume (24). Pfu Rnl and the fusion protein were loaded separately on the column and elution volumes were recorded for molecular weight comparison (25).

### Standard reaction conditions

The standard phosphorylation reactions were performed according to the manual for the commercial T4 PNK (New England Biolabs). The standard adenylation reactions were carried out in a 20-μl reaction mixture containing 70 mM Tris–HCl buffer with pH 7.0, 5 mM dithiothreitol (DTT), 100 μM ATP, 5 mM MgCl_2_, 5 μM phosphorylated oligonucleotides and 5 μM Pfu Rnl. The reaction mixtures were incubated in the S1000 Thermal Cycles (Bio-Rad) at 80°C for 30 min. After adding 20 μl of 2× formamide loading buffer (95% formamide, 18 mM EDTA, 0.025% SDS, Bromophenol Blue and Xylene Cyanol FF), the products were denatured at 95°C for 5 min, separated by electrophoresis with a 20% denaturing polyacrylamide gel containing 7 M urea in 1× TBE, then stained with SYBR Gold (Invitrogen) and visualized using the ChemiDoc Imaging System (Bio-Rad). The extent of adenylation was quantified using Quantity One software [intensity of adenylated band/(intensity of phosphorylated band + intensity of adenylated band)]. All assays were performed in triplicate. Modifications to standard reaction conditions were made as specified. Adenylation reactions using the 5′ DNA adenylation kit (New England Biolabs) were performed according to the manufacturer's protocol. The adenylated products were purified using the Oligo Clean & Concentrator kit (Zymo Research). Mass spectrometry analyses were performed on the MassARRAY Mass Spectrometry System (Agena Bioscience) according to the manufacturer's protocol (26).

A 55-nt oligonucleotide with 5′ phosphorylation (pDNA55g) was used for adenylation efficiency comparison between Pfu Rnl and Mth Rnl (NEB). The adenylation products were analyzed with urea–PAGE. To confirm that the adenylated product can be further used for ligation, the adenylation product of Pfu Rnl was circularized with CircLigase™ ssDNA ligase (Lucigen) without ATP. The circularized product was then treated with exonuclease I (NEB).

For the adenylation and ligation test, a 17-nt oligonucleotide was used. The 5′-phosphorylated DNA or RNA oligonucleotide was incubated with equal molar amounts of Pfu Rnl or Mth Rnl at 37, 65 or 80°C with or without ATP. All of the products were analyzed with urea–PAGE.

### Combination of T4 PNK and Pfu Rnl for adenylation

Standard reaction mixtures (20 μl) containing 70 mM Tris–HCl (pH 7.0), 5 mM DTT, 10 mM MgCl_2_, 100 μM ATP, 5 pmol of 5′-hydroxyl oligonucleotides, 5 pmol of T4 PNK and 10 pmol of Pfu Rnl were incubated at 80°C for 30 min. Reactions were terminated by adding 20 μl of 2× formamide loading buffer. Products were resolved on denaturing PAGE gel as described above.

### Direct adenylation using fusion enzymes

Standard reaction mixtures (20 μl) contained 5 pmol of fusion protein with 70 mM Tris–HCl (pH 7.0), 5 mM DTT, 10 mM MgCl_2_, 100 μM ATP and 5 pmol oligonucleotide. Assays were performed using a thermocycler (Bio-Rad) under the conditions described in the figure legends. The products were resolved on denaturing gel and by mass spectrometry as described above and in the figure legends.

## RESULTS

### Sequence alignment and structure prediction

**Figure 1.**
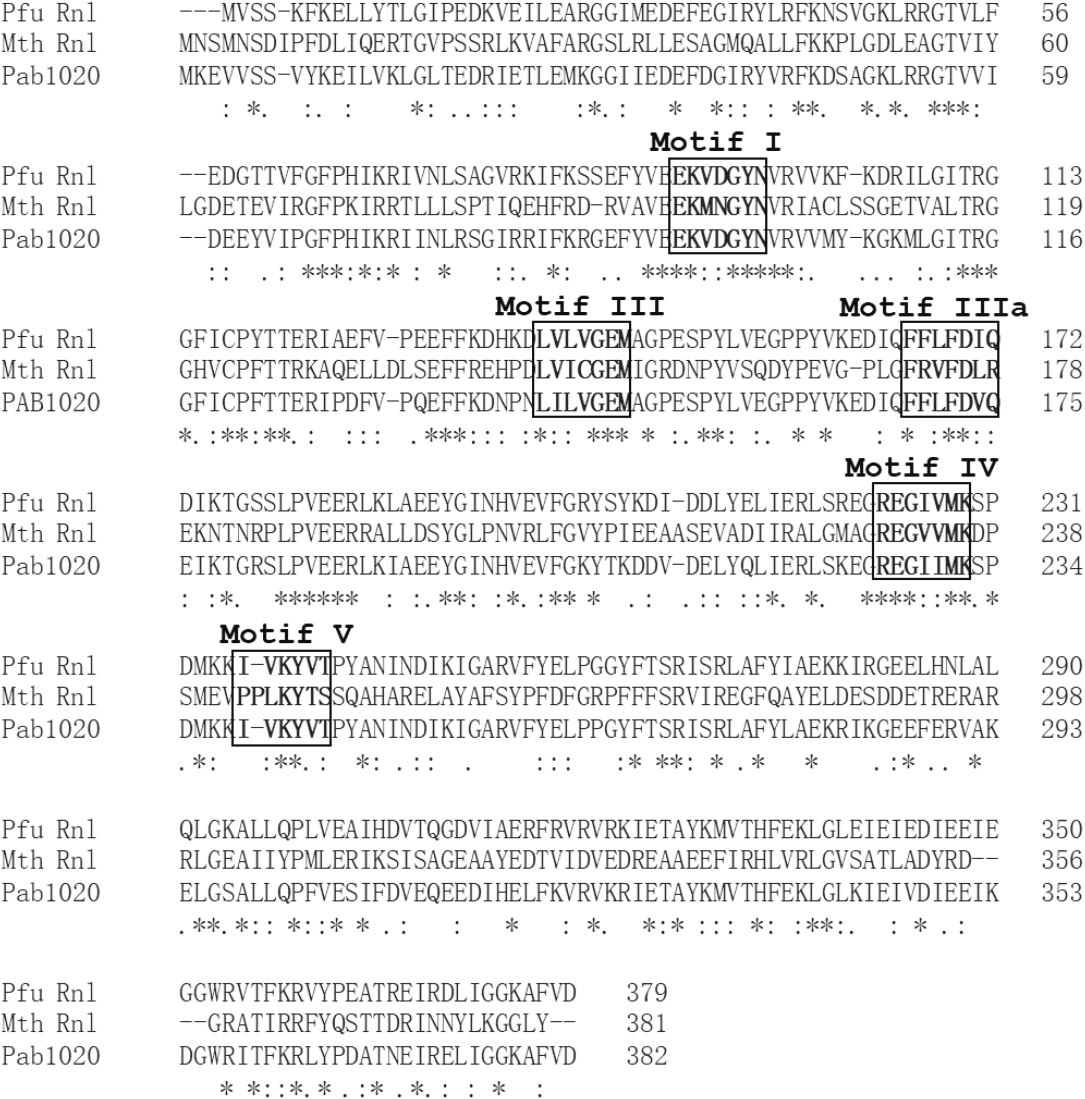
Sequence comparison of Pfu RNA ligase (Pfu Rnl), Mth RNA ligase (Mth Rnl) and Pab1020 protein. The motifs I, III, IIIa, IV and V are labeled in bold and boxed. The asterisk represents identical residues. The : symbol represents amino acids with similar properties. The alignment was made with the Clustal Omega online software.

**Figure 2.**
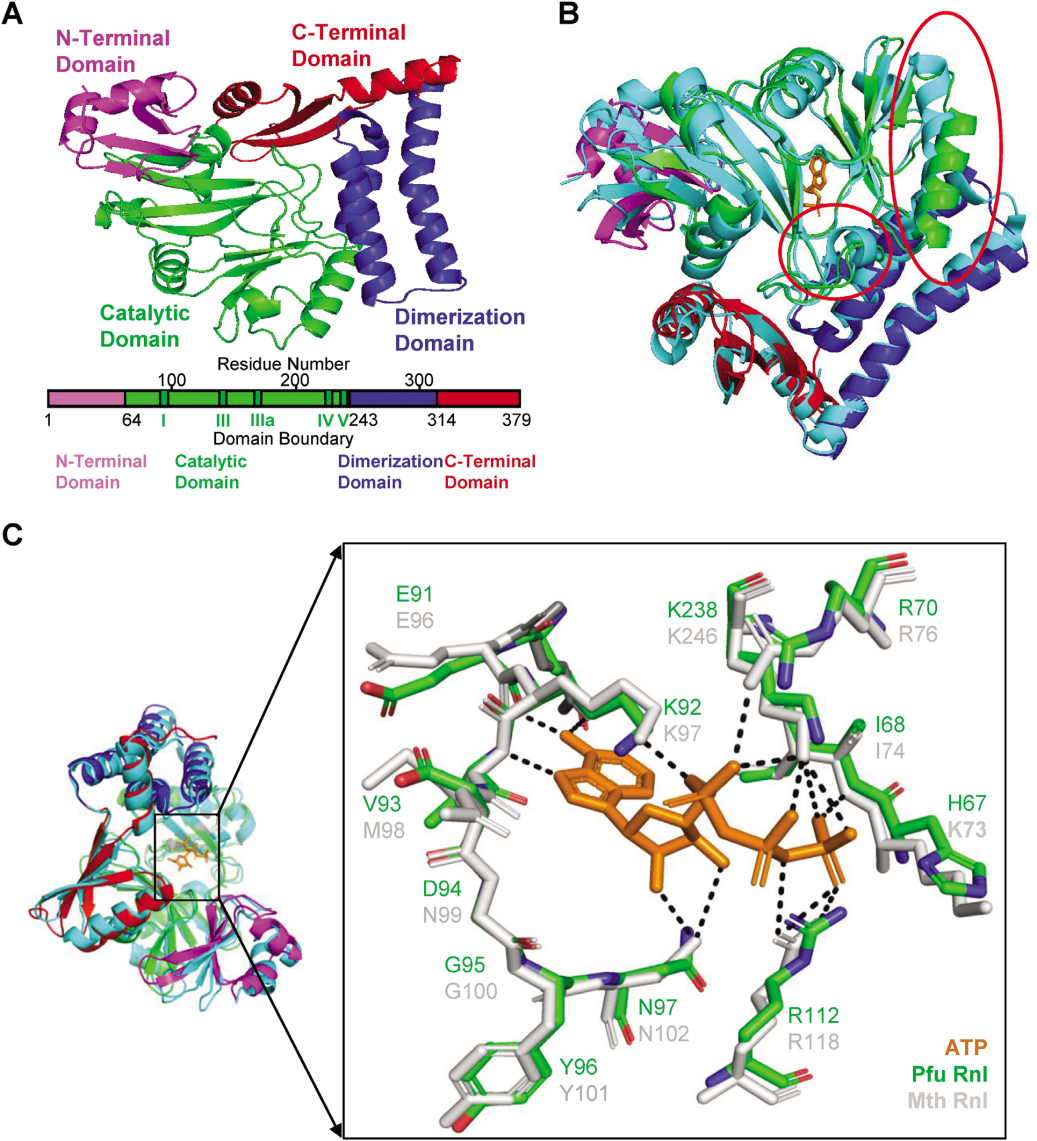
Predicted structure of Pfu Rnl and comparison with Mth Rnl. (**A**) The four domains of Pfu Rnl are highlighted with different colors. Schematic diagram of the domain boundaries is shown below, with conserved motifs I, III, IIIa, IV and V of the nucleotidyl transferase superfamily shown in dark green. (**B**) Structure superposition of Pfu Rnl and Mth Rnl. Among the few noticeable structural differences, the red circle in the middle highlights the α-helix missing in Pfu Rnl. The red circle in the top right corner highlights a shortened α-helix in Pfu Rnl. (**C**) The superposed structure of the ATP-binding pocket in Pfu Rnl and Mth Rnl. The hydrogen bonds are shown by dotted lines.

Through sequence homology analysis (27), we identified that the Q8U3V2 protein (hereafter named Pfu Rnl) encoded by the *PF0353* gene of the hyperthermophilic archaea *Pyrococcus furiosus* contains motifs homologous to Mth Rnl, a commercially available adenylation enzyme (16), and Pab1020, a member of the RNA ligase 3 family (18) (Figure 1). We predicted the structure of Pfu Rnl using the Swiss-Model database by its sequence homology to Pab1020. The predicted Pfu Rnl consists of four structural domains including an N-terminal domain, a catalytic domain, a dimerization domain and a C-terminal domain (Figure 2A). The predicted structure of Pfu Rnl is similar to the structure of Mth Rnl. The main structural differences were located in the N-terminal domain and the catalytic domain. Pfu Rnl has one less α-helix and shorter α-helices and β-sheets in the catalytic domain as compared with Mth Rnl (Figure 2B). The dimerization domain was almost identical to that of Mth Rnl, suggesting that Pfu Rnl may form homodimers (Figure 2B). Pfu Rnl showed a similar ATP-binding pocket to Mth Rnl, except that Met98 and Lys73 of Mth Rnl were replaced with Val93 and His67, respectively (Figure 2C). The hydrogen bonds were similarly formed for these two amino acid positions.

### Expression, purification and characterization of the Pfu RNA ligase

The full-length *PF0353* gene is 1140 bp, coding for Pfu Rnl with 379 amino acids. The recombinant Pfu Rnl was produced as a His-tagged protein in *E. coli* (Supplementary Figure S1). The His-tagged protein with a molecular weight of ∼42 kDa was purified to ∼95% purity as estimated by Commassie Blue staining (Supplementary Figure S1A). Gel filtration showed that Pfu Rnl was mainly a homodimer, consistent with the structural predictions (Supplementary Figure S1B).

A series of oligonucleotides were used in various assays. For simplicity, we used the short name OH-DNA for 5′-OH terminal DNA oligonucleotide, pDNA for 5′-phosphorylated terminal DNA oligonucleotide, AppDNA for 5′-adenylated terminal DNA oligonucleotide, OH-RNA for 5′-OH terminal RNA oligonucleotide, pRNA for 5′-phosphorylated terminal RNA oligonucleotide and AppRNA for 5′-adenylated terminal RNA oligonucleotide.

**Figure 3.**
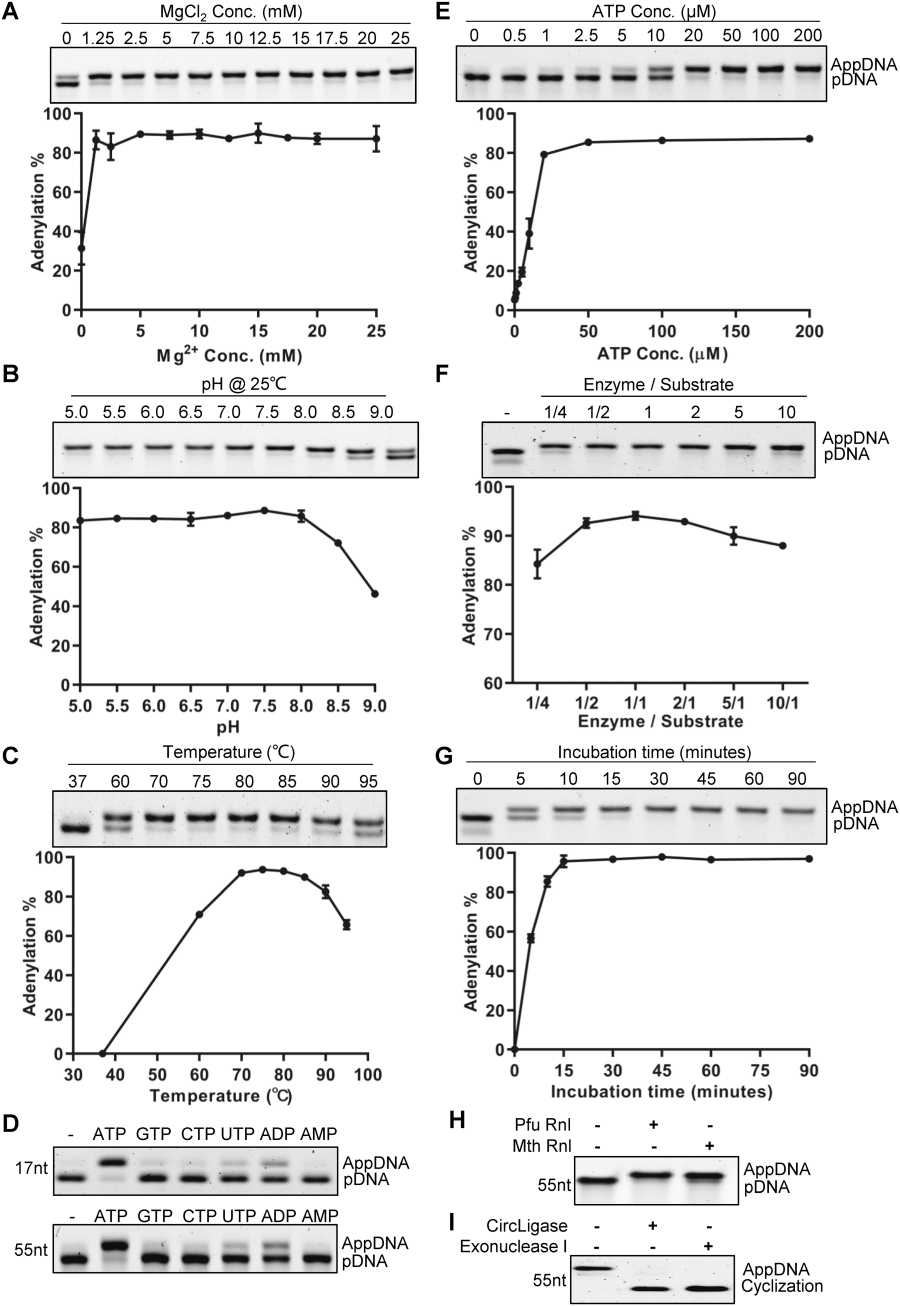
Characterization of Pfu RNA ligase activity. (**A**) Mg^2+^ concentration. (**B**) pH in Tris–HCl buffer. (**C**) Temperature. (**D**) Mononucleotides. The assays were performed with 17-nt and 55-nt ssDNAs. (**E**) ATP concentration. (**F**) Enzyme to substrate ratios. (**G**) Incubation time. (**H**) The adenylation activity of Pfu Rnl and Mth Rnl was compared using phosphorylated oligonucleotide. (**I**) The adenylated product was circularized with CircLigase ssDNA ligase without ATP and treated with exonuclease I. Error bars represent the standard deviations of three independent experiments.

To characterize the recombinant Pfu Rnl, we performed conversion assays using 5′ phosphorylated substrates (16). The optimum ion was determined to be the divalent magnesium ion (data not shown). Titration assays showed that maximum conversion efficiency was achieved with 5 mM magnesium chloride (Figure 3A). Pfu Rnl has a broad pH window with optimum pH between 5 and 8 in 70 mM Tris–HCl buffer with 5 mM magnesium chloride (Figure 3B). Thus, a reaction buffer containing 70 mM Tris–HCl and 5 mM magnesium chloride at pH 7.0 was used as the standard reaction buffer for Pfu Rnl. The enzyme is thermostable, with the best temperature window from 70 to 85°C, with ∼60% catalytic activity at 95°C (Figure 3C). Pfu Rnl showed the best activity with ATP as the adenosyl source, weak catalytic activity with ADP and almost no catalytic activity with AMP (Figure 3D). Pfu Rnl showed optimum activity with 50–200 μM ATP (Figure 3E).

We next investigated the catalytic efficiency with different enzyme:substrate (E/S) ratios. When the E/S ratio was 1/4, ∼84% of the substrates were adenylated. The adenylation efficiency was highest with E/S ratios between 1/2 and 2 (Figure 3F). Our results were different from what was reported for Mth Rnl where an E/S ratio of 1:1 was required for efficient adenylation (16). We thus performed parallel experiments for Pfu Rnl and Mth Rnl, using a 1:5 E/S ratio. With prolonged incubation, Pfu Rnl was able to almost completely adenylate the phosphorylated substrate at 120 min, whereas with Mth Rnl a substantial amount of substrate remained unconverted at 120 min (Supplementary Figure S2). At a 1:1 E/S ratio, a time course experiment showed that adenylation was completed after 15–30 min (Figure 3G).

To further validate that the presumed adenylated product is suitable for downstream applications, we performed adenylation with a 55-nt 5′ phosphorylated oligonucleotide using Pfu Rnl and Mth Rnl. As expected, both enzymes achieved near complete conversion (Figure 3H). The adenylated molecule produced by Pfu Rnl was successfully circularized by CircLigase, a ssDNA ligase which specifically circularizes adenylated ssDNA without ATP (20), as demonstrated by survival after exonuclease I digestion (Figure 3I).

**Figure 4.**
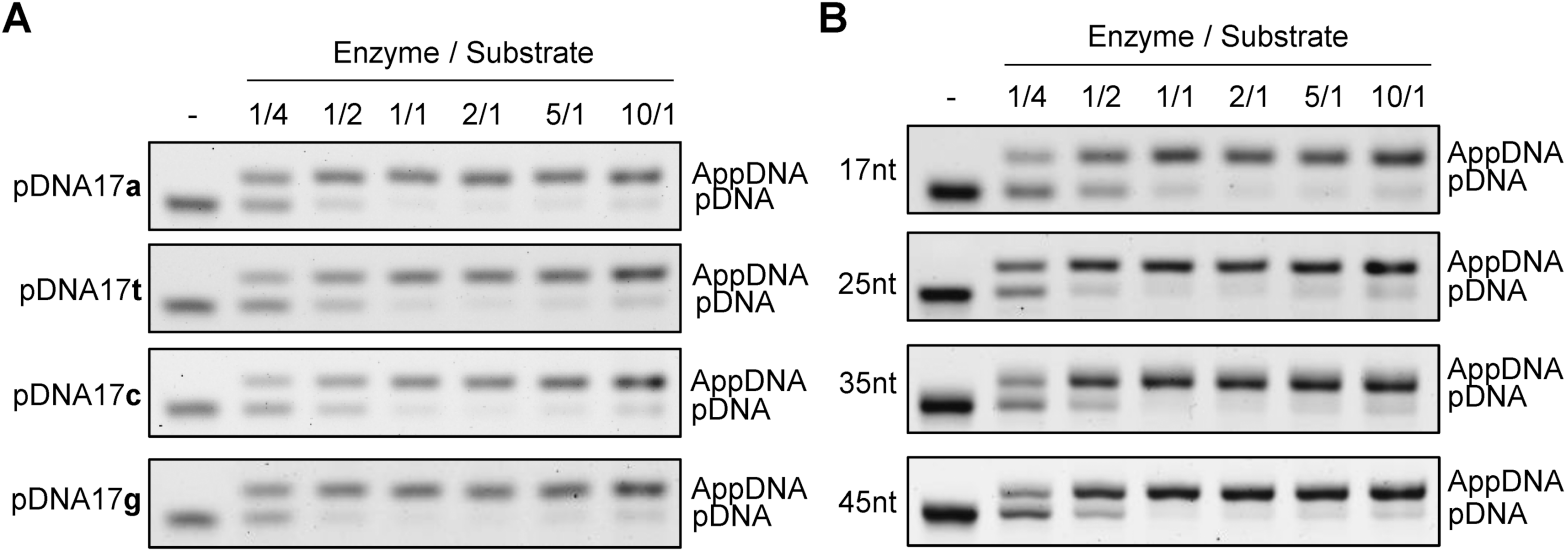
Effect of the substrate 5′ nucleotide and DNA length on the efficiency of adenylation. (**A**) Adenylation efficiency was evaluated using 17-nt oligonucleotides with identical sequences except at the 5′-phosphorylated terminus. Substrates were catalyzed under various enzyme to substrate ratios as specified. (**B**) Adenylation efficiency was evaluated using 17-, 25-, 35- or 45-nt phosphorylated oligonucleotides and under various enzyme to substrate ratios.

To determine whether the substrate end sequence may affect the catalytic activity of Pfu Rnl, we tested adenylation using oligonucleotides with different 5′ end nucleotides. At an equimolar amount, the adenylation reactions were near complete, irrespective of the 5′ end nucleotide (Figure 4A). Adenylation reactions were also near complete with oligonucleotides ranging from 17 to 45 nt (Figure 4B). These results demonstrated that Pfu Rnl can efficiently adenylate 5′-phosphorylated oligonucleotides, irrespective of their length and 5′ terminal nucleotide sequence.

### Ligation properties of Pfu RNA ligase

We next tested the ligation activities of Pfu Rnl. Pfu Rnl efficiently circularized a 5′-phosphorylated RNA oligonucleotide in the absence of ATP at 80°C (Supplementary Figure S3A). However, the presence of 100 μM ATP inhibited substrate circularization (Supplementary Figure S3A). For comparison, Mth Rnl generated both linear ligation and circularization products in the absence of ATP (Supplementary Figure S3A). Similarly, ATP inhibited ligations. For 5′-phosphorylated DNA oligonucleotides, neither Mth Rnl or Pfu Rnl showed ligation activity (Supplementary Figure S3B).

### Converting the 5′-OH terminal oligonucleotide to the adenylated product with T4 PNK and Pfu Rnl in a single reaction

As 5′-OH terminal oligonucleotides (5′-OH-Oligos) are most routinely produced at a low cost, we reasoned that a protocol to convert 5′-OH-Oligos directly to adenylated forms may be preferred. T4 PNK is known to phosphorylate 5′-OH-Oligos in the presence of ATP (Supplementary Figure S4A). We thus tested whether T4 PNK and Pfu Rnl can be used in a single reaction and with a common buffer condition to sequentially convert 5′-OH-Oligos to phosphorylated and finally to adenylated products (Supplementary Figure S4A). Indeed, when both enzymes were incubated in the standard Pfu Rnl reaction buffer, the 5′-OH-Oligo was almost fully converted to the adenylated product when the E/S ratio was set at 2/1 (Supplementary Figure S4B, last lane). Due to the thermostability of T4 PNK, the reactions were first set at 37°C to perform the phosphorylation step, followed by 80°C to perform adenylation.

### Engineering of T4 PNK and Pfu Rnl fusion enzymes for direct conversion of 5′-OH terminal DNA oligonucleotides to the adenylated form

Encouraged by the compatibility of T4 PNK and Pfu Rnl in a single reaction, we tested whether fusion proteins of these two enzymes may possess both phosphorylation and adenylation activity, thereby providing a single enzyme to directly catalyze 5′-OH terminal substrates to adenylated products.

**Figure 5.**
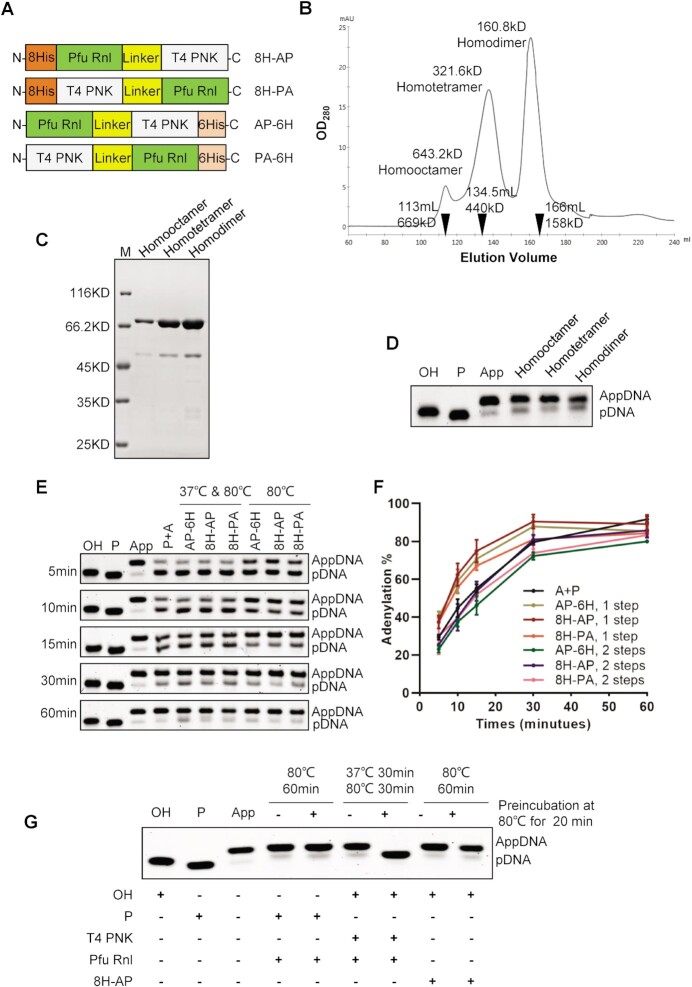
Fusion proteins with T4 PNK and Pfu Rnl directly convert a hydroxy substrate to an adenylated product. (**A**) Schematic diagram of four different fusion proteins with T4 PNK and Pfu Rnl. 8His (8H) and 6His (6H) stand for His tags. The linker is a GS amino acid repeat polypeptide. The short names of the fusion enzymes are specified on the right. (**B**) Gel filtration results for the fusion protein 8H-AP. The molecular weights and elution volumes of the protein standards are marked as inverted triangles. The presumed homodimer, homotetramer and homo-octamer were further analyzed by SDS–PAGE (**C**) and enzymatic reactions (**D**). (**E**) A time-series experiment with different enzyme/enzyme combinations. P + A stands for the 5′-OH-terminated oligonucleotide treated with T4 PNK and Pfu Rnl in a two-step reaction as a control, with a quantitative analysis shown in (**F**). (**G**) Demonstration of acquired thermostability for T4 PNK in the fusion enzyme via pre-incubation of 80°C for 20 min.

Fusion proteins were produced with a GS-rich linker as described previously (20) between the two enzymes (Figure 5A). We designed four types of fusion proteins for expression in *E. coli* (Supplementary Figure S5A). Three fusion proteins were successfully expressed and purified (Supplementary Figure S5B). When analyzed by gel filtration, fusion protein 8H-AP showed three forms with molecular weights consistent with a homodimer, homotetramer and homo-octamer, respectively (Figure 5B). The three forms were further analyzed by SDS–PAGE, demonstrating they were all formed by Pfu Rnl monomer (Figure 5C). Additionally, all three forms were active for adenylation (Figure 5D).

Using an OH-DNA, the three fusion proteins all generated adenylation products in a two-step temperature reaction, 37°C for T4 PNK and 80°C for Pfu Rnl (Figure 5E and F). Surprisingly, the fusion enzymes were able to perform the full reaction at a single temperature of 80°C (Figure 5E and F). We speculated that somehow engineering the fusion enzymes may have unexpectedly improved the thermostability of T4 PNK. To test this, we heated the fusion enzyme 8H-AP at 80°C for 20 min prior to the adenylation reaction. The OH-DNA was still successfully adenylated to near completeness (Figure 5G). As a control, adenylation failed when T4 PNK and Pfu Rnl were pre-heated (lane 7, Figure 5G), consistent with what is known about the thermostability of T4 PNK (28).

To assess more quantitatively the completeness of adenylation, we performed mass spectrometric analysis of the reaction products (Supplementary Figure S6). Pfu Rnl was able to convert 95.4% of 5′-phosphorylated substrate to the adenylated form. Combination of two separate enzymes (T4 PNK and Pfu Rnl) was able to convert 92.1% of the 5′-OH terminal substrate to the adenylated form, while the conversion completeness of the 8H-AP fusion enzyme was 91.3% (Supplementary Figure S6).

Similar to Pfu Rnl alone, the 8H-AP fusion enzyme was able to convert OH-DNA to near completeness with prolonged incubation, even when the fusion enzyme was added at an E/S ratio of 1:5 (Supplementary Figure S2).

The 8H-AP fusion enzyme has much lower activity to convert 5′-OH terminal RNA, even when the fusion enzyme was present at a 6:1 E/S ratio (Supplementary Figure S7A). This low activity may be due to lower conversion from phosphorylated to adenylated forms as 8H-AP also had poor activity on 5′-phosphorylated RNA (Supplementary Figure S7B). As Pfu Rnl alone was able to convert pRNA to AppRNA with high efficiency (Supplementary Figure S3A, last lane). We speculated that there might be some structural changes in the fusion protein, resulting in reduced activity.

## DISCUSSION

5′-Adenylated oligonucleotides (AppOligos) have been widely used in various reactions including ssDNA/RNA ligation-based NGS applications such as microRNA sequencing (1–3). The adenylated adapter removes the need to dephosphorylate DNA/RNA targets, simplifying library preparation while also eliminating self-ligation. ssDNA ligation-based NGS analysis using adenylated adapters may also be advantageous for analyzing DNA samples with low quality, such as DNA derived from formalin-fixed paraffin-embedded (FFPE) tissues samples or cell-free DNA from blood samples (29–32).

We characterized Pfu Rnl, a novel thermostable enzyme from *Pyrococcus furiosus* with catalytic activity to convert 5′-phosphorylated DNA or RNA oligonucleotides to adenylated products. Pfu Rnl shares sequence and structural similarities with Mth Rnl. Interestingly, Pfu Rnl is more thermostable (Figure 5G). Mth Rnl is inactivated at 85°C for 5 min, while Pfu Rnl is fully active at 80°C, with >60% activity at 95°C (Figure 3C).

We also found that T4 PNK and Pfu Rnl share a compatible reaction buffer (Supplementary Figure S4B). To further facilitate the production of AppOligos, we engineered fusion enzymes of Pfu Rnl and T4 PNK which can directly convert 5′-OH terminal substrates to adenylated forms. The 8H-AP fusion protein forms homodimer, homotetramer and homo-octamer structures (Figure 5B and C). As T4 PNK forms homotetramers (33–35) and Archaeal RNA ligases including Pfu Rnl form homodimers (18,19,36), we speculate that the various homo-oligomers may be formed by either T4 PNK or Pfu Rnl. We confirmed that all forms of homo-oligomers were catalytically active.

Interestingly, the fusion enzymes were able to complete both phosphorylation and adenylation at a single reaction temperature of 80°C, suggesting that T4 PNK may have unexpectedly acquired thermostability in the fusion proteins (Figure 5E and F). We confirmed the acquired thermostability of T4 PNK by showing full activity of the 8H-AP enzyme after pre-incubation at 80°C (Figure 5G).

There is also room for further research and improvement. Firstly, the fusion enzymes did not fully convert hydroxy substrates to adenylated products (Figure 5E and F). This is probably due to the incomplete adenylation by the Pfu Rnl activity, as mass spectrometric analysis showed phosphorylated intermediates while hydroxy substrates were fully consumed (Supplementary Figure S6). Thus, further directed mutagenesis and reaction condition optimization may be needed to enhance the Pfu Rnl activity. It will also be interesting to further analyze the mechanism underlying how T4 PNK, via fusing with a thermostable Pfu Rnl, acquired thermostability.

In summary, Pfu Rnl and the fusion proteins may provide useful tools to study DNA modifications and to produce AppOligos. A one-step conversion from 5′-OH-Oligo to AppOligo by a T4 PNK and Pfu Rnl fusion enzyme may greatly simplify AppOligo production at a reduced cost. We estimate the consumable cost for a small-scale production sufficient for 20 ligations is ∼US$1, which is significantly cheaper than commercial AppOligo chemical synthesis at >US$1000. Thus, Pfu Rnl and the fusion enzyme may offer a cost-effective alternative for small research laboratories using AppOligos for relevant applications.

## DATA AVAILABILITY

Fusion protein sequences described in this work are listed in Supplementary Table S3.

## Supplementary Material

gkac604_Supplemental_File
