# Supplementary material for: Direct adenylation from 5′-OH-terminated oligonucleotides by a fusion enzyme containing Pfu RNA ligase and T4 polynucleotide kinase
Source: Nucleic Acids Res. 2022 Jul 12;50(13):7560–9. doi: 10.1093/nar/gkac604 (PMC9303275; doi:10.1093/nar/gkac604)
Supplement: gkac604_Supplemental_File [file gkac604_supplemental_file.pdf]

SUPPORTING INFORMATION

Direct adenylation from 5’-OH terminated oligonucleotides by a fusion enzyme containing Pfu RNA ligase and T4 polynucleotide kinase

Zhengquan Yang[1,†], Chengliang Zhang[1,2, †], Guojun Lian[1], Shijie Dong[1], Menghui Song[1], Hengrong Shao[1], Jingmei Wang[1], Tao Zhong[1], Zhenni Luo[1], Shengnan Jin[1,*] and Chunming Ding[1,*]

[1] Key Laboratory of Laboratory Medicine, Ministry of Education of China, School of Laboratory Medicine and Life Science, Wenzhou Medical University, Wenzhou, Zhejiang, 325035, China
[2] Department of Clinical Laboratory, Kunming Third People's Hospital, Kunming, Yunnan, 650041, China

* To whom correspondence should be addressed. Tel: +86 577 86689190; Fax: +86 577 86689779; Email: cmding@gmail.com

Correspondence may also be addressed to Shengnan Jin. Email: snjin1997@qq.com

[†]The authors wish it to be known that, in their opinion, the first two authors should be regarded as joint First Authors

TABLE AND FIGURES LEGENDS

Supplementary Table 1. Oligonucleotide sequence and modifications

Name	Sequence
pDNA17a	5´-/5Phos/ATGTAGGCACCATCAAT-3´
pDNA17t	5´-/5Phos/TTGTAGGCACCATCAAT-3´
pDNA17c	5´-/5Phos/CTGTAGGCACCATCAAT-3´
pDNA17g	5´-/5Phos/GTGTAGGCACCATCAAT-3´
pDNA25g	5´-/5Phos/GTGTAGGCACCATCAATAGTGAATT-3´
pDNA35g	5´-/5Phos/GTGTAGGCACCATCAATAGTGAATTCGAGCTCGGT-3´
pDNA45g	5´-/5Phos/GTGTAGGCACCATCAATAGTGAATTCGAGCTCGGTACC CGGTGGA-3´
pDNA55g	5´-/5Phos/GTGTAGGCACCATCAATAGTGAATTCGAGCTCGGTACC CGGTGGATCCTCTAGAG-3´
DNA55g	5´-GTGTAGGCACCATCAATAGTGAATTCGAGCTCGGTACCCGGTG GATCCTCTAGAG-3´
AppDNA55g	5´-/5rApp/GTGTAGGCACCATCAATAGTGAATTCGAGCTCGGTACC CGGTGGATCCTCTAGAG-3´
DNA17g	5´-GTGTAGGCACCATCAAT-3´
DNA17t	5´-TTGTAGGCACCATCAAT-3´
RNA17u	5´-UUGUAGGCACCAUCAAU-3´
pRNA17u	5´-/5Phos/UUGUAGGCACCAUCAAU-3´

Supplementary Table 2. Oligonucleotide sequence for fusion protein plasmid construction

Name	Sequence
PNK_ins_F	AGCGGTGGCAGTGGCGGTAGCGGTGGCAGTGCGGGTATGAAGAAGATAATTCTGACCA
PNK_ins_R	TGGTGGTGGTGCTCGAGTTAGTCGCCGCTCGCCACCTG
Pfu_F	TAACTCGAGCACCACCACCACCACCACTGAGATC
Pfu_R	CTACCGCCACTGCCACCGCTACTATCAACAAAGGCTTTACCACCAA

Supplementary Table 3. Names and sequences of fusion proteins

Name	Sequence
8H-AP	MHHHHHHHHENMVSSKFKELLYTLGIPEDKVEILEARGGIMEDEFEGIRYLRFKNSVGKLRRGTVLFEDGTTVFGFPHIKRIVNLSAGVRKIFKSSEFYVEEKVDGYNVRVVKFKDRILGITRGGFICPYTTERIAEFVPEEFFKDHKDLVLVGEMAGPESPYLVEGPPYVKEDIQFFLFDIQDIKTGSSLPVEERLKLAEEYGINHVEVFGRYSYKDIDDLYELIERLSREGREGIVMKSPDMKKIVKYVTPYANINDIKIGARVFYELPGGYFTSRISRLAFYIAEKKIRGEELHNLALQLGKALLQPLVEAIHDVTQGDVIAERFRVRVRKIETAYKMVTHFEKLGLEIEIEDIEEIEGGWRVTFKRVYPEATREIRDLIGGKAFVDSSGGSGGSGGSAGKKIILTIGCPGSGKSTWAREFIAKNPGFYNINRDDYRQSIMAHEERDEYKYTKKKEGIVTGMQFDTAKSILYGGDSVKGVIISDTNLNPERRLAWETFAKEYGWKVEHKVFDVPWTELVKRNSKRGTKAVPIDVLRSMYKSMREYLGLPVYNGTPGKPKAVIFDVDGTLAKMNGRGPYDLEKCDTDVINPMVVELSKMYALMGYQIVVVSGRESGTKEDPTKYYRMTRKWVEDIAGVPLVMQCQREQGDTRKDDVVKEEIFWKHIAPHFDVKLAIDDRTQVVEMWRRIGVECWQVASGD
8H-PA	MHHHHHHHHKKIILTIGCPGSGKSTWAREFIAKNPGFYNINRDDYRQSIMAHEERDEYKYTKKKEGIVTGMQFDTAKSILYGGDSVKGVIISDTNLNPERRLAWETFAKEYGWKVEHKVFDVPWTELVKRNSKRGTKAVPIDVLRSMYKSMREYLGLPVYNGTPGKPKAVIFDVDGTLAKMNGRGPYDLEKCDTDVINPMVVELSKMYALMGYQIVVVSGRESGTKEDPTKYYRMTRKWVEDIAGVPLVMQCQREQGDTRKDDVVKEEIFWKHIAPHFDVKLAIDDRTQVVEMWRRIGVECWQVASGDSSGGSGGSGGSAGENMVSSKFKELLYTLGIPEDKVEILEARGGIMEDEFEGIRYLRFKNSVGKLRRGTVLFEDGTTVFGFPHIKRIVNLSAGVRKIFKSSEFYVEEKVDGYNVRVVKFKDRILGITRGGFICPYTTERIAEFVPEEFFKDHKDLVLVGEMAGPESPYLVEGPPYVKEDIQFFLFDIQDIKTGSSLPVEERLKLAEEYGINHVEVFGRYSYKDIDDLYELIERLSREGREGIVMKSPDMKKIVKYVTPYANINDIKIGARVFYELPGGYFTSRISRLAFYIAEKKIRGEELHNLALQLGKALLQPLVEAIHDVTQGDVIAERFRVRVRKIETAYKMVTHFEKLGLEIEIEDIEEIEGGWRVTFKRVYPEATREIRDLIGGKAFVD
AP-6H	MENMVSSKFKELLYTLGIPEDKVEILEARGGIMEDEFEGIRYLRFKNSVGKLRRGTVLFEDGTTVFGFPHIKRIVNLSAGVRKIFKSSEFYVEEKVDGYNVRVVKFKDRILGITRGGFICPYTTERIAEFVPEEFFKDHKDLVLVGEMAGPESPYLVEGPPYVKEDIQFFLFDIQDIKTGSSLPVEERLKLAEEYGINHVEVFGRYSYKDIDDLYELIERLSREGREGIVMKSPDMKKIVKYVTPYANINDIKIGARVFYELPGGYFTSRISRLAFYIAEKKIRGEELHNLALQLGKALLQPLVEAIHDVTQGDVIAERFRVRVRKIETAYKMVTHFEKLGLEIEIEDIEEIEGGWRVTFKRVYPEATREIRDLIGGKAFVDSSGGSGGSGGSAGKKIILTIGCPGSGKSTWAREFIAKNPGFYNINRDDYRQSIMAHEERDEYKYTKKKEGIVTGMQFDTAKSILYGGDSVKGVIISDTNLNPERRLAWETFAKEYGWKVEHKVFDVPWTELVKRNSKRGTKAVPIDVLRSMYKSMREYLGLPVYNGTPGKPKAVIFDVDGTLAKMNGRGPYDLEKCDTDVINPMVVELSKMYALMGYQIVVVSGRESGTKEDPTKYYRMTRKWVEDIAGVPLVMQCQREQGDTRKDDVVKEEIFWKHIAPHFDVKLAIDDRTQVVEMWRRIGVECWQVASGDHHHHHH
PA-6H	MKKIILTIGCPGSGKSTWAREFIAKNPGFYNINRDDYRQSIMAHEERDEYKYTKKKEGIVTGMQFDTAKSILYGGDSVKGVIISDTNLNPERRLAWETFAKEYGWKVEHKVFDVPWTELVKRNSKRGTKAVPIDVLRSMYKSMREYLGLPVYNGTPGKPKAVIFDVDGTLAKMNGRGPYDLEKCDTDVINPMVVELSKMYALMGYQIVVVSGRESGTKEDPTKYYRMTRKWVEDIAGVPLVMQCQREQGDTRKDDVVKEEIFWKHIAPHFDVKLAIDDRTQVVEMWRRIGVECWQVASGDSSGGSGGSGGSAGENMVSSKFKELLYTLGIPEDKVEILEARGGIMEDEFEGIRYLRFKNSVGKLRRGTVLFEDGTTVFGFPHIKRIVNLSAGVRKI

	FKSSEFYVEEKVDGYNVRVVKFKDRILGITRGGFICPYTTERIAEFVPEEFFKDHKDL<br>VLVGEMAGPESPYLVEGPPYVKEDIQFFLFDIQDIKTGSSLPVEERLKLAEEYGINHV<br>EVFGRYSYKDIDDLYELIERLSREGREGIVMKSPDMKKIVKYVTPYANINDIKIGARVF<br>YELPGGYFTSRISRLAFYIAEKKIRGEELHNLALQLGKALLQPLVEAIHDVTQGDVIAE<br>RFRVRVRKIETAYKMVTHFEKLGLEIEIEDIEEIEGGWRVTFKRVYPEATREIRDLIGG<br>KAFVDHHHHHH

A

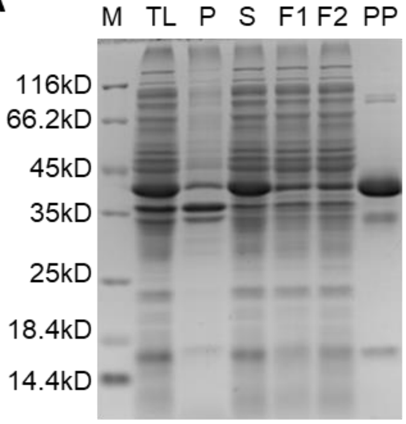

B

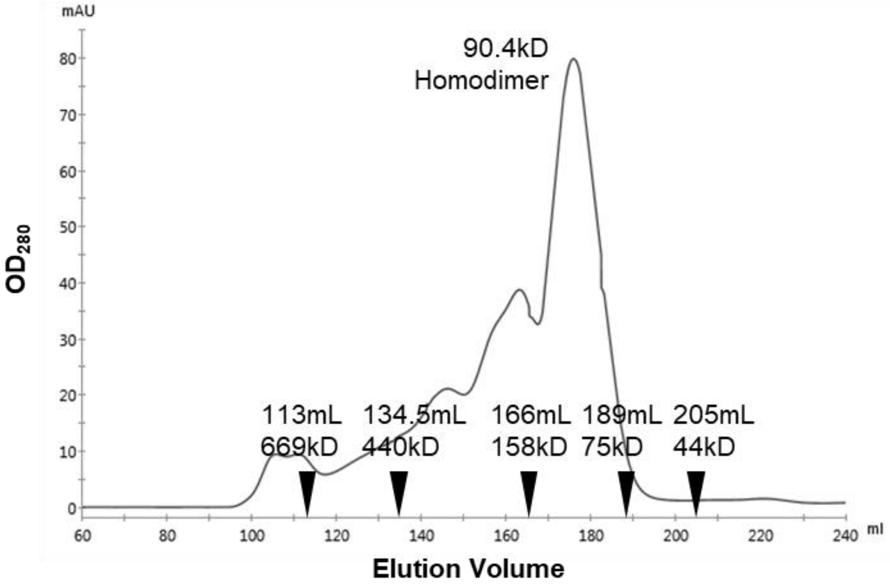

Supplementary Figure 1. Purification of the 8×His-tagged recombinant Pfu Rnl. (A) SDS-PAGE analysis of the recombinant Pfu Rnl. M, protein molecular weight ladder; TL, total lysate; P, precipitation after lysate centrifugation; S, supernatant; F1 and F2, flowthrough 1 and 2; PP, purified protein. (B) Gel filtration of the purified Pfu Rnl. Molecular weights and elution volume of standard proteins were marked.

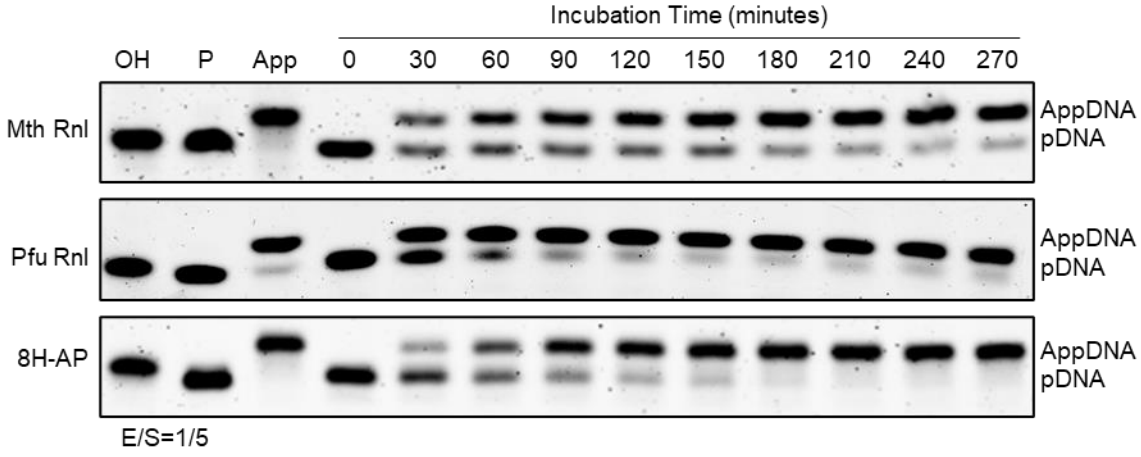

Supplementary Figure 2. Adenylation efficiency analysis with reduced enzyme amount and prolonged incubation. Reduced enzyme (Mth Rnl, Pfu Rnl, or 8H-AP) to substrate ratio of 1:5 and longer incubation times were used. A 5’ phosphorylated substrate (pDNA17t) was used for Mth Rnl and Pfu Rnl. A 5’ OH terminal substrate (DNA17t) was used for the 8H-AP fusion protein.

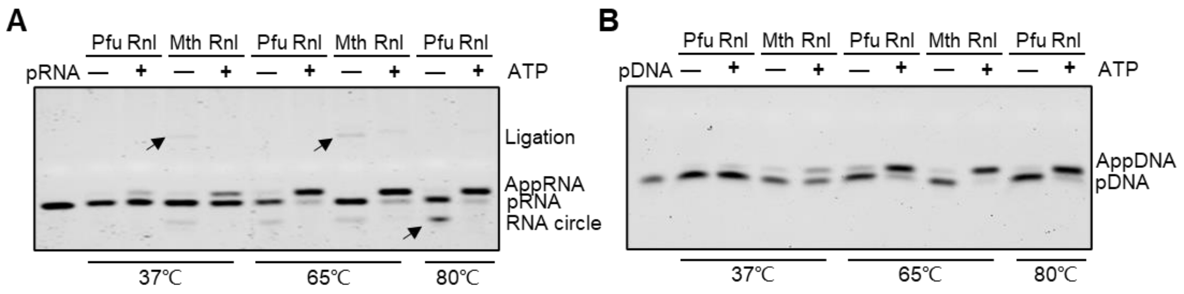

Supplementary Figure 3. DNA and RNA adenylation and ligation activities of Pfu Rnl with different modified substrates and temperatures. (A) pRNA (pRNA17u), (B) pDNA (pDNA17t) were used as substrate. The linear or cyclization products were marked. Mth Rnl was also analyzed as a control.

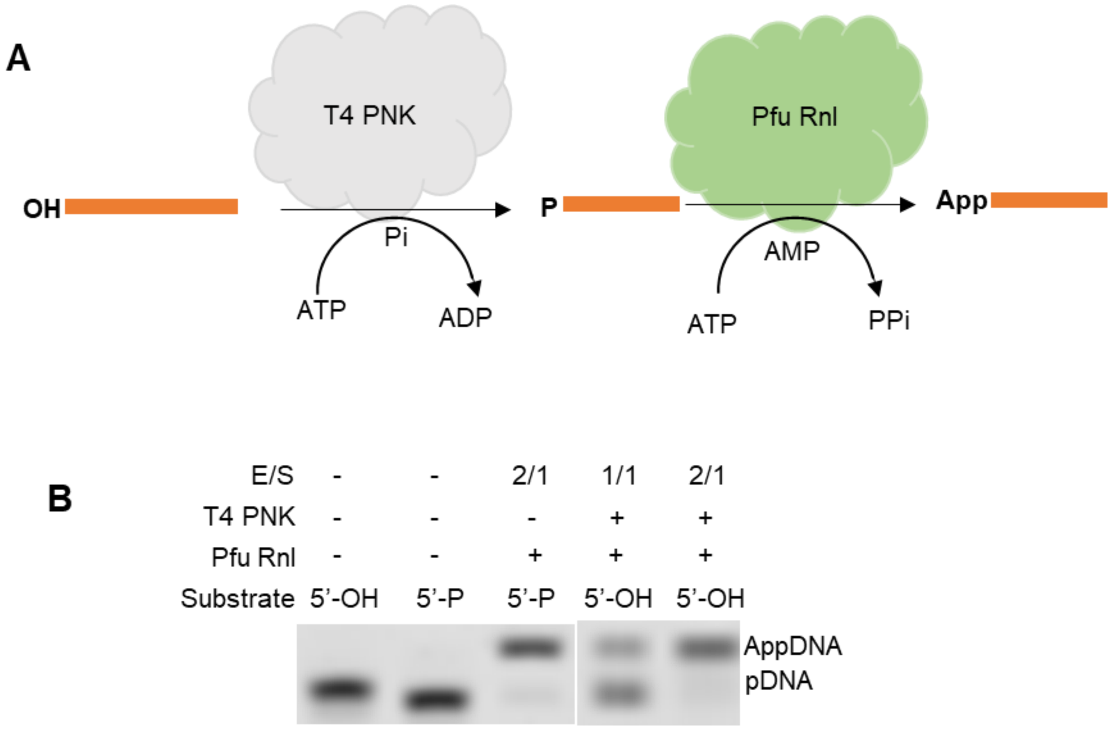

Supplementary Figure 4. Combination T4 PNK and Pfu Rnl directly converted a OH-DNA to an adenylated product. (A) Schematic diagram of combination of T4 PNK and Pfu Rnl in one assay to directly convert a OH-DNA to App-DNA. (B) Adenylation by combination of T4 PNK and Pfu Rnl using OH-DNA or pDNA substrate. E/S, enzyme : substrates ratio.

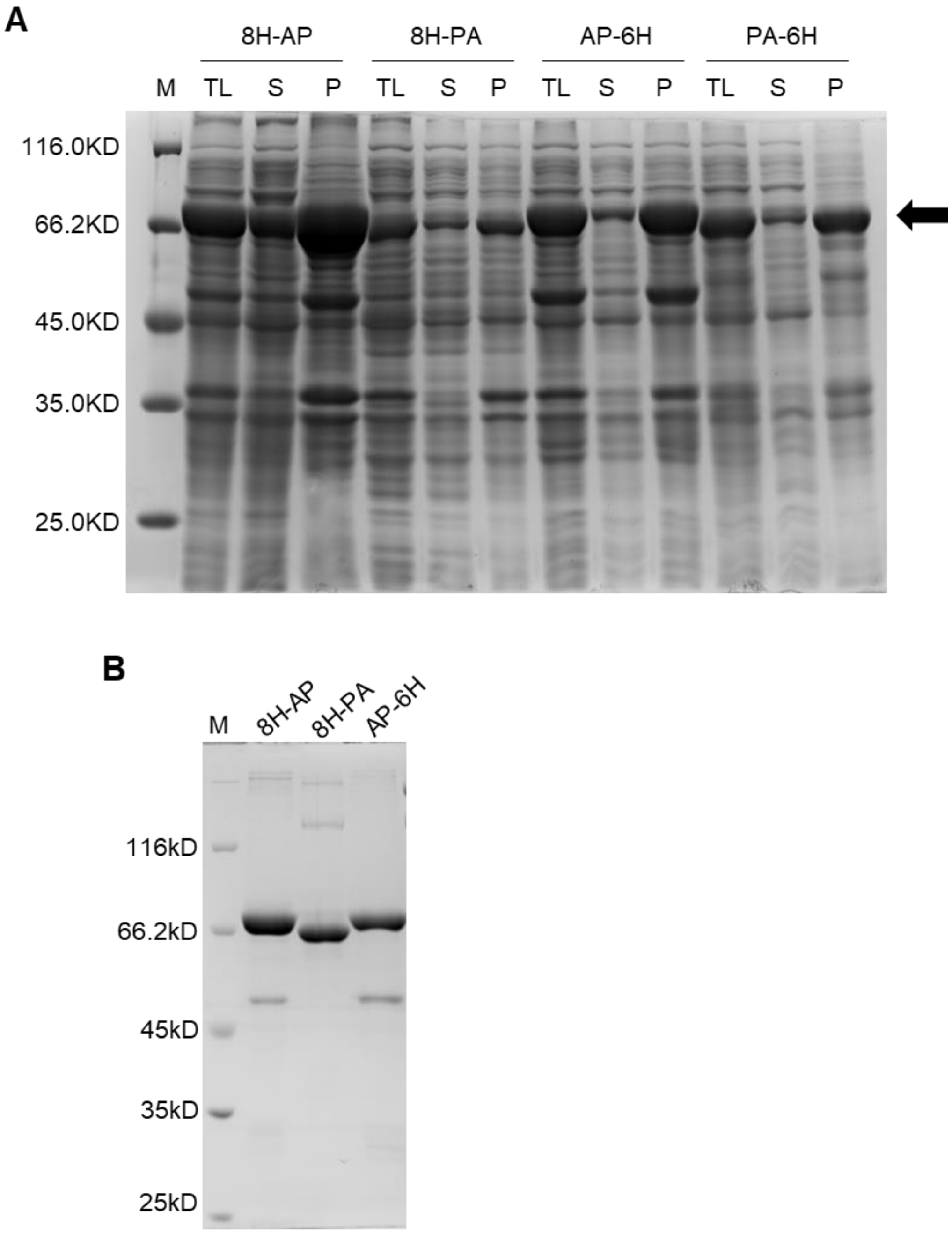

Supplementary Figure 5. Purification of the His-tagged recombinant fusion protein. (A) SDS-PAGE analysis of the recombinant fusion proteins. M, protein molecular weight ladder; TL, total lysate; S, supernatant; P, precipitation. (B) Purified fusion proteins.

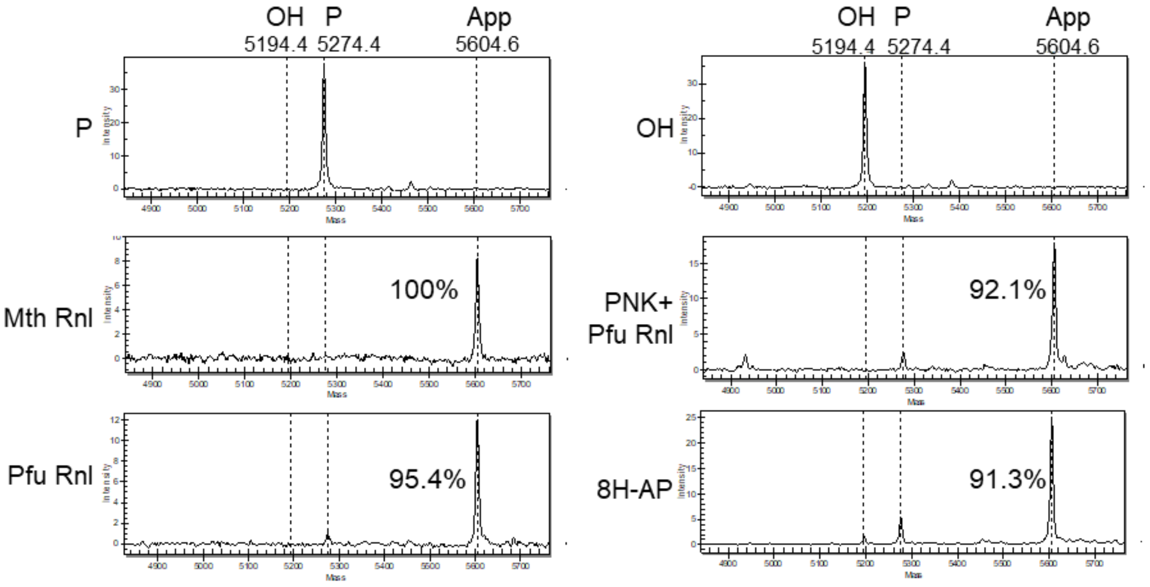

Supplementary Figure 6. Quantitative adenylation analysis using MALDI-TOF-MS. Adenylation completeness of Mth RNA ligase (Mth Rnl), Pfu RNA ligase (Pfu Rnl), T4 PNK and Pfu Rnl (two-step reaction with different incubation temperatures), and fusion enzyme 8H-AP were quantified using MALDI-TOF-MS. For Mth Rnl and Pfu Rnl, the pDNA was used as the substrate. For the other two reactions, OH-DNA17 was used as the substrate. OH, P and App stand for 5'-OH terminal, 5'-phosphorylated and 5'-adenylated oligonucleotides. The molecular weights of the oligonucleotides were marked above the mass spectra. The percentages of the adenylated product after each reaction were also provided.

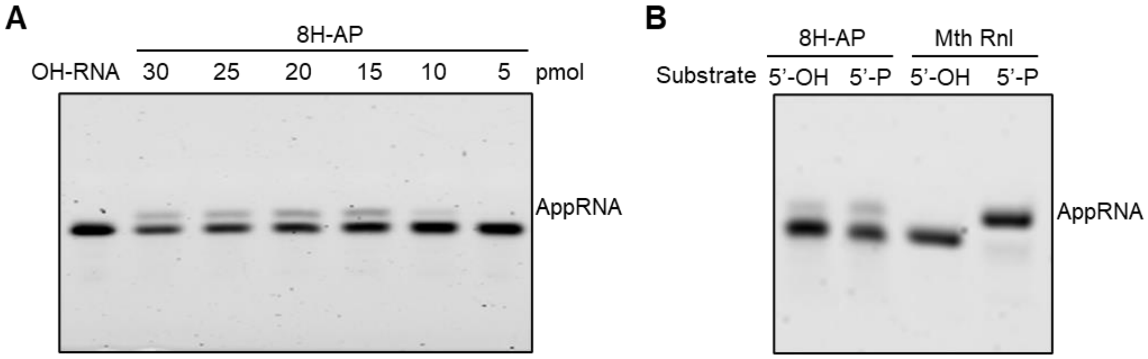

Supplementary Figure 7. Fusion protein has weak adenylation activity with 5-OH and 5-phosphorylation terminated RNA oligonucleotides. (A) OH-RNA adenylation assays (5 pmol) with different amounts of 8H-AP fusion protein. (B) OH-RNA (5'-OH) or pRNA (5'-P) adenylation assays with 10 pmol fusion protein and 1 pmol RNA substrate.
